# Preventing the inappropriate treatment of asymptomatic bacteriuria at a community teaching hospital

**DOI:** 10.3402/jchimp.v2i2.17814

**Published:** 2012-07-16

**Authors:** Farhana Chowdhury, Kumkum Sarkar, Angela Branche, Juliette Kim, Philip Dwek, Angelica Nangit, David Tompkins, Ernest Visconti

**Affiliations:** 1 Department of Internal Medicine, Lutheran Medical Center, New York, NY, USA; 2 St. George's University School of Medicine, True Blue, Grenada, West Indies; 3 Department of Pharmacy, Lutheran Medical Center, New York, NY, USA

**Keywords:** practice guidelines as topic, guideline adherence, trends, urinary tract infections, economics, prevention and control, disease management

## Abstract

The goal of this study was to assess the overtreatment of asymptomatic bacteriuria (ASB) in hospitalized patients, calculate the total costs of inappropriate treatment, and determine if a multi-faceted educational intervention was effective in reducing the overtreatment of ASB in a resource-limited community hospital. The study encompassed three phases: a retrospective pre-intervention assessment of the baseline cost and treatment of ASB, the implementation of a multi-faceted educational intervention, and a prospective post-intervention assessment of the efficacy of the intervention. A positive urine culture was defined by bacterial counts ≥10^5^ cfu/mL. In the pre-intervention group, 64 (83%) of 109 patients were asymptomatic: 30 (47%) were treated. In the post-intervention group, 13 (17%) of 55 patients were asymptomatic: 2 (15%) were treated, (*p*=0.04). Fewer urine cultures were collected during the post-intervention period than the pre-intervention period (3,127 and 3,419, respectively) (*p*<0.001). The total cost of inappropriately treating ASB in the pre-intervention group was $1200 compared to $600 in the post-intervention group. The results demonstrated a significant decrease in the inappropriate treatment of ASB and the associated costs.

Treatment of asymptomatic bacteriuria (ASB) is a common practice seen in various institutionalized settings (1, 2). Current guidelines recommend that selected patients, including pregnant women and those undergoing surgical urologic interventions, benefit from screening and treatment of ASB (3, 4). In all other cases, however, literature suggests that the role of antibiotic therapy may be limited in providing clinical benefit (5–9). It can, in fact, lead to potential harm, as it allows for the emergence of resistant bacterial strains, and development of adverse drug reactions and *Clostridium difficile* causing pseudomembranous colitis (1–2, 10). All of these factors can result in increased hospital acquisition costs (1, 2, 7, 11). By targeting the initial screening and diagnostic approach, antimicrobial usage and hospital resources can be preserved.

Institutions around the country have utilized various approaches to address the overtreatment of ASB. One institution (2) proposed the development of a hospital and ambulatory performance measure for not treating ASB in adults; whereas another institution (7) used an educational memorandum based on guideline recommendations for appropriate diagnosis and treatment. The electronic memorandum was inserted into the charts of patients who were diagnosed with ASB and receiving antimicrobial therapy. This led to a 65% relative reduction in the number of days that antimicrobials were used (11). Other studies have been conducted to address treatment in specific sub-sets of the population. These studies individually discuss treatment in pregnant women, diabetics, spinal cord injury patients, urological surgery candidates, and institutionalized patients (3–9). Our study is unique in that it describes an educational intervention to decrease the overtreatment of ASB in a resource-limited community teaching hospital.

The goal of our study was to assess the overtreatment of ASB in hospitalized patients, calculate the total costs of inappropriate treatment, and determine if a multi-faceted educational intervention was effective in reducing the overtreatment of ASB in a resource-limited community hospital.

## Methods

This study encompassed three phases: a retrospective pre-intervention assessment of the baseline cost and treatment of ASB, the implementation of a multi-faceted educational intervention, and a prospective post-intervention assessment of the efficacy of the intervention.

### Pre-intervention

A retrospective chart review examined medical records for inpatient and nursing home patients with positive urine cultures for the month of February 2010. Patients included in this portion of the study were older than 18 years, had no signs or symptoms of a urinary tract infection (UTI), and had a urine culture with bacterial counts of ≥10^5^ cfu/mL. Patients with spinal cord injury and catheterized patients while the catheter remains *in situ* were also included. Pregnant women, patients with co-morbidities requiring antibiotic treatment, or patients undergoing urologic procedures with a high risk of mucosal bleeding were excluded. If a patient was diagnosed with ASB based on the research tool and received subsequent treatment, the cost was calculated based on the pre-interventional per-patient antibiotic cost compared to the post-interventional cost.

### Intervention

#### Clinical vignettes

In January 2011, a hospital-wide intervention was initiated. This intervention included an educational seminar where six clinical vignettes were presented in a live session using a real-time audience response system. The presentation highlighted guideline recommendations and recent literature findings pertaining to ASB management. In addition, the clinical vignettes addressed common clinical issues faced in most heath care facilities, including ASB in young patients with spinal cord injuries and chronic indwelling catheters, pregnant women, and diabetics, and the role of antibiotic prophylaxis in patients undergoing urologic procedures. It also highlighted the importance of not treating ASB in institutionalized elderly women, elderly patients with multiple co-morbidities, and patients with indwelling catheters. Finally, antibiotic choices based on history of previous susceptibility and co-morbidities were discussed.

#### Pocket cards

The intervention also included pocket cards which highlighted the following Infectious Diseases Society of America (IDSA) guidelines: ASB defined as two consecutive voided urine specimens with isolation of the same organism in quantitative counts ≥10^5^ cfu/mL in women with no signs or symptoms of UTI, or a single, clean catch voided urine specimen with one bacterial species isolated in quantitative count ≥10^5^ cfu/mL without any signs or symptoms of UTI in men (1). In both men and women, a single catheterized urine specimen with one bacterial species isolated in a quantitative count ≥10^2^ cfu/mL was defined as bacteriuria (1).

**Fig. 1:**
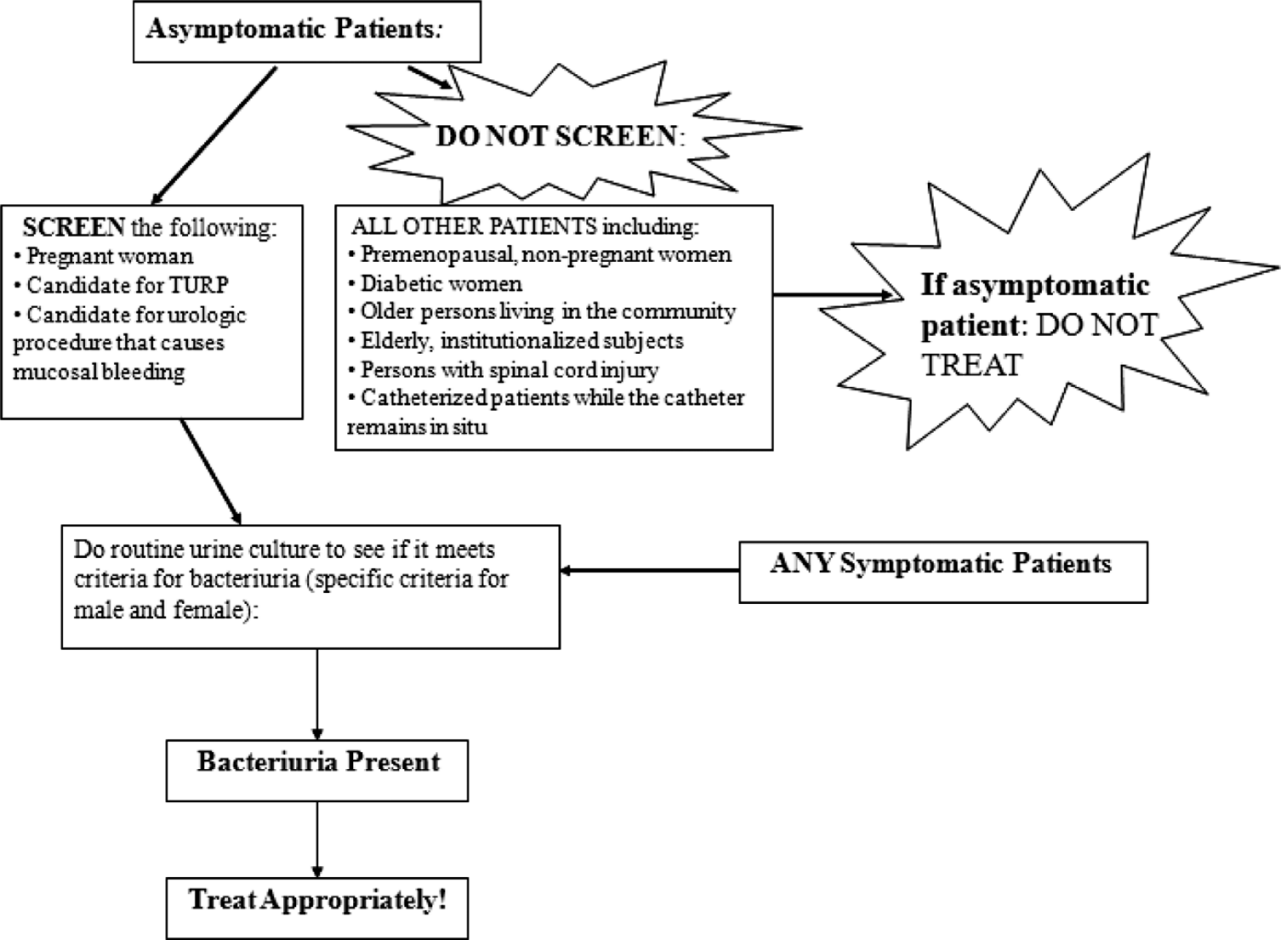
Front of pocket card.

**Fig. 2:**
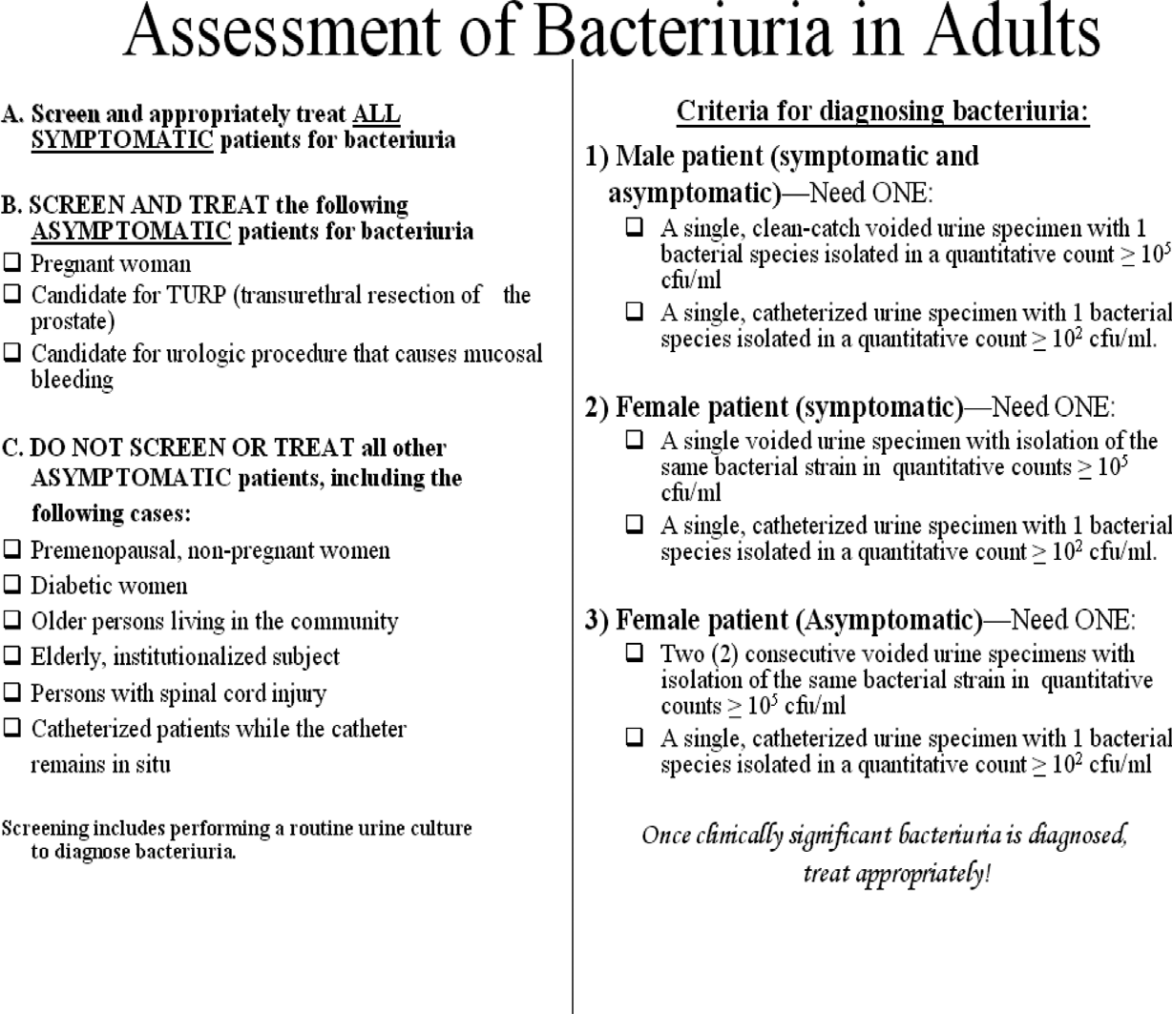
Back of pocket card.

The pocket cards were 6×6 inches in size, where one side included an algorithm of when to screen and treat asymptomatic patients (Fig. 1). The reverse side included IDSA guidelines and criteria for the diagnosis of bacteriuria (Fig. 2). The pocket cards were distributed to the Internal Medicine, OB/GYN, Surgery, and Family Medicine departments, followed by a brief presentation of the results from the pre-intervention phase and the goal of the study.

#### Promotional letter

The last intervention included a letter sent to the hospital attending physician staff to promote awareness of the IDSA guideline recommendations and the hospital wide intervention. It also emphasized the importance of minimizing unnecessary antimicrobial exposure to prevent bacterial resistance.

### Post-intervention

Post-intervention data collection took place from February 14 to March 15, 2011. Charts of patients with positive urine cultures, defined as bacterial counts greater than 10^5^ cfu/mL, were reviewed for documentation of symptoms of a UTI, including fever, urgency, frequency, dysuria, suprapubic tenderness, and change in mental status. Patients who were pregnant, had co-morbid infections requiring antibiotic treatment, or underwent urologic procedures with high risk or mucosal bleeding were excluded.

### Data analysis

The proportion of patients with positive urine cultures who were inappropriately treated in the pre-intervention period was compared to the post-intervention period using the chi-square test. Group differences were compared using independent sample *t*-tests or chi-square tests. The pharmacy department calculated the total per-patient cost of antibiotics for inappropriately treating ASB, which were compared pre-intervention and post-intervention.

## Results

Demographic characteristics of both groups are reported in Table 1.

**Table 1:** Pre- and post-intervention patient population characteristics

	Pre-intervention	Post-intervention	*p*-Value
Average age (years)	76.2 (±12.92)	74.40 (±15.94)	0.435
Population			
Female	68%	71%	
Male	32%	29%	0.725
Foley catheter			
Yes	51%	55%	
No	49%	45%	0.742
Trauma patients with Foley catheter			
Yes	1%	6%	
No	99%	94%	0.224
Diabetic			
Yes	40%	42%	
No	60%	58%	0.868
Immunocompromised			
Yes	4%	7%	
No	96%	93%	0.444
Infection at other sites			
Yes	44%	49%	
No	56%	51%	0.619
Symptomatic			
Yes	41%	76%	
No	59%	24%	<0.001

### Pre-intervention

Of 109 patients with positive urine cultures, 64 (59%) had ASB and 45 (41%) were diagnosed with symptomatic bacteriuria. Of the 64 asymptomatic patients with positive urine cultures, 30 (47%) were inappropriately treated: 29 of those 30 patients were hospital inpatients. Furthermore, 54% of catheterized patients and 42% of non-catheterized patients were inappropriately treated (*p*=0.344). The total calculated cost of over-treating 30 patients was $1200.

### Post-intervention

In the post-intervention phase, 55 patients had positive urine cultures, 13 (24%) patients were asymptomatic and 42 (76%) were diagnosed with symptomatic bacteriuria. Two (15%) of the asymptomatic patients were inappropriately treated for bacteriuria. This demonstrates a significant decrease in the proportion of inappropriately treated patients (*p*=0.036). Fewer urine cultures were collected during the post-intervention period than the pre-intervention period (3,127 and 3,419, respectively) (*p*<0.001). The total cost of inappropriately treating ASB in the in the post-intervention group was $600.

## Discussion

Our findings demonstrated that our educational interventions reduced hospital costs and the overtreatment of ASB in a resource-limited community teaching hospital. Various intervention tools and strategies have been utilized in hospitals worldwide. Among the many include reference cards, Infectious Disease physician lectures, electronic memorandums, and advice-based discussions that aim at preventing overtreatment of ASB (11–15). Other studies have shown results similar to ours: an improvement in the management of ASB demonstrated by a reduction in antimicrobial usage, number of urine cultures sent, and number of asymptomatic patients over treated for bacteriuria (12–20).

The findings in our study are also comparable to studies utilizing both educational and non-educational interventions (11–15, 18–20). Our study, like others, involved pre- and post-intervention assessments of inappropriate treatment (11, 14). Furthermore, the studies strived to maintain similar pre- and post-intervention populations in terms of their baseline characteristics, and had similar exclusion criteria as to which sub-sets of the population should be treated for ASB (1). For example, Linares et al. (11) considered patients to be asymptomatic for UTI if another process more appropriately explained the documented symptom. We also excluded individuals with co-morbid infection, in order to avoid the difficulty in assessing the source of infection (11).

### Limitations

One of the main limitations of our research was a lack of differences in the types of patients in our sample. Pregnant women, patients undergoing urologic procedures, and patients with spinal cord injuries were not represented among our post-intervention study sample. We were also limited by our method of data collection, which was manual chart review. Currently, patient medical records at our institution are hand written by attending physicians and residents. Clinically significant findings, such as UTI symptoms, may not have been clearly documented in the patient's medical record. In addition, UTI symptoms may have varied in presentation among different patient populations, particularly in the elderly, where non-specific symptoms may be suggestive of another source of infection. Excluding patients with other documented underlying infections generally avoided this complication. Unlike some of the other educational intervention studies with more than 115 patients in their sample (15, 20), our sample size was much smaller. Despite this, significant reductions in inappropriate treatment were observed.

We also observed a decrease in the number of urine cultures sent, although we did not calculate the cost associated with this decrease. Therefore, the reported cost savings with the overtreatment of ASB is likely underestimated given that the dollar amount reflects direct medication costs. We would expect the savings to be higher if we incorporated other costs related to management and treatment, including urine cultures and urinalysis.

Finally, our post-intervention group was not as large as our pre-intervention group of patients. Although we saw a decrease in the proportion of patients treated with ASB, it is difficult to clearly assess this decrease due to the difference in the size of the pre- and post-intervention groups. The smaller post-intervention patient population can be attributed to the fact that the intervention phase did not include nursing home patients, unlike the patient populations used in other studies (13, 16, 19). Therefore, we utilized percentages to note our changes and improvements. On the other hand, the greater number of asymptomatic patients in the pre-intervention group compared to the post-intervention group may suggest that the educational interventions also decreased inappropriate screening of asymptomatic patients. Further, the prevalence of symptomatic bacteriuria was similar in the pre-intervention (45 patients/3,419 urine cultures, 1.3%) and post-intervention (42 patients/3,127 urine cultures, 1.3%) demonstrating that the patient populations were similar.

### Future studies

Further studies warrant a larger sample size and longer time frame for data collection in order to better assess the effects of our intervention and demonstrate its sustainability (12). Although our study showed an improvement in preventing the overtreatment of ASB during the post-intervention period, we did not assess the reason for overtreatment in patients such as diabetics and elderly institutionalized individuals with co-morbidities. Studies assessing the rationale for treating ASB in such patients may help address this issue.

## Conclusions

Our results demonstrated that a multi-faceted educational intervention successfully reduced the overtreatment of ASB and its associated costs in a resource-limited community teaching hospital. Because our intervention was implemented at a low cost, it should be sustainable and reproducible in other institutions.
